# Acyl-CoA Synthetase 5 Knockout and Inhibitors Protect Against Diet-Induced Obesity in Mice by Activating the Ileal Brake

**DOI:** 10.1210/jendso/bvaf196

**Published:** 2025-11-28

**Authors:** David R Powell, Isaac Van Sligtenhorst, Alan Main, Haihong Jin, Kenneth G Carson, Zhi-Cai Shi, Jonathan Swaffield, Melinda Smith, Angela Harris, Suma Gopinathan, Kenneth A Platt, Jeffrey Wade, Brian Zambrowicz, Patricia McDonald, Darren Orton, Lakshmi Kuttippurathu, Michael Mullens, Jennifer Greer, Qi Melissa Yang, Zhi-Ming Ding

**Affiliations:** Lexicon Pharmaceuticals, The Woodlands, TX 77381, USA; Lexicon Pharmaceuticals, The Woodlands, TX 77381, USA; Lexicon Pharmaceuticals, The Woodlands, TX 77381, USA; Lexicon Pharmaceuticals, The Woodlands, TX 77381, USA; Lexicon Pharmaceuticals, The Woodlands, TX 77381, USA; Lexicon Pharmaceuticals, The Woodlands, TX 77381, USA; Lexicon Pharmaceuticals, The Woodlands, TX 77381, USA; Lexicon Pharmaceuticals, The Woodlands, TX 77381, USA; Lexicon Pharmaceuticals, The Woodlands, TX 77381, USA; Lexicon Pharmaceuticals, The Woodlands, TX 77381, USA; Lexicon Pharmaceuticals, The Woodlands, TX 77381, USA; Lexicon Pharmaceuticals, The Woodlands, TX 77381, USA; Lexicon Pharmaceuticals, The Woodlands, TX 77381, USA; Lexicon Pharmaceuticals, The Woodlands, TX 77381, USA; Lexicon Pharmaceuticals, The Woodlands, TX 77381, USA; Lexicon Pharmaceuticals, The Woodlands, TX 77381, USA; Lexicon Pharmaceuticals, The Woodlands, TX 77381, USA; Lexicon Pharmaceuticals, The Woodlands, TX 77381, USA; Lexicon Pharmaceuticals, The Woodlands, TX 77381, USA; Lexicon Pharmaceuticals, The Woodlands, TX 77381, USA

**Keywords:** gene knockout, small molecule, inhibitor, obesity, energy intake, insulin sensitivity

## Abstract

Genes regulating body fat are shared by mice and humans, and mouse knockout phenotypes for known drug targets correlate well with drug efficacy, suggesting that mouse knockout phenotyping can identify anti-obesity drug targets. Mice with an intestine-specific *Acsl5* knockout are protected from high-fat diet (HFD)-induced obesity, insulin resistance, glucose intolerance and hepatic steatosis, and show increased GLP-1 levels, delayed gastric emptying (GE), and decreased food consumption (FC). Here we provide data on these and further outcomes in mice with a global *Acsl5* knockout and in mice receiving ACSL5 inhibitors (ACSL5i). We generated *Acsl5* knockout mice by homologous recombination and identified potent ACSL5i by compound library screening, iterative medicinal chemistry optimization, and by testing whether compounds inhibit oral triglyceride absorption. We found that both genetic and pharmacologic ACSL5 inhibition reproduce the intestine-specific knockout metabolic phenotype. Importantly, the ACSL5i LP-856866 lowered FC in wild-type but not *Acsl5* knockout mice, indicating targeted ACSL5 inhibition. *Acsl5* knockout mice had increased fecal free fatty acids but not triglycerides, and adding the lipase inhibitor orlistat to an oral triglyceride load reversed the delayed GE associated with genetic and pharmacologic ACSL5 inhibition; these findings, and the marked GLP-1 release after mice with genetic and pharmacologic ACSL5 inhibition received an oral triglyceride load, suggest ileal brake activation. We conclude that HFD-fed *Acsl5* knockout mice exhibit a favorable metabolic phenotype, driven by ileal brake activation, which is phenocopied by orally available small molecule ACSL5i.

Study Importance Questions:

What is already known about this subject?
  - Male mice with an intestine-specific knockout (KO) of *Acsl5* are protected from high-fat diet (HFD)-induced obesity, insulin resistance, glucose intolerance, and hepatic steatosis and show increased GLP-1 levels, delayed gastric emptying (GE), decreased food consumption (FC), and decreased serum triglyceride (TG) and total cholesterol levels.
What are the new findings in your manuscript?
  - Male and female mice with a global *Acsl5* KO reproduced the favorable metabolic phenotype described in HFD-fed male mice with an intestine-specific KO, and, in addition, had minimal loss of lean body mass, which is a desirable trait in a weight loss target.
  - We developed small molecule ACSL5 inhibitors (ACSL5i) which, when delivered orally to HFD-fed mice, reproduced the favorable metabolic phenotype found in HFD-fed *Acsl5* KO mice.
  - Our ACSL5i LP-856866 lowered HFD FC in wild-type but not in *Acsl5* KO mice, indicating that LP-856866 targets ACSL5.
  - HFD-fed *Acsl5* KO mice have elevated intestinal free fatty acids (FFAs). When intestinal FFA release is prevented by blocking TG digestion in HFD-fed mice with genetic or pharmacologic ACSL5 inhibition, GE is no longer delayed. This suggests that HFD intake by ACSL5-deficient mice triggers the ileal brake mechanism, resulting in the favorable metabolic phenotype observed.
How might your results change the direction of research or the focus of clinical practice?
  - Mouse gene KO phenotypes model with high fidelity: (i) the phenotype of humans deficient in activity of the same gene; and (ii) the phenotype of humans treated with inhibitors of the protein product of this gene.
  - Because the fidelity of this modeling is particularly high for metabolic phenotypes, the favorable metabolic phenotype of HFD-fed mice with genetic or pharmacologic *Acsl5* inhibition suggests that ACSL5i might be effective and desirable anti-obesity therapeutics in humans.

The obesity pandemic is a major public health problem because obesity is the common denominator behind the pathophysiological interrelatedness of diabetes, cardiovascular disease, and chronic kidney disease that together create a profound health and economic burden [1, 2]. Recently developed glucagon-like peptide 1 receptor (GLP-1R) and GLP-1R/gastric inhibitory polypeptide (GIPR) agonists may control this pandemic by their ability to induce marked body weight (BW) loss that lessens the severity of obesity-related comorbidities [3, 4]. These incretin mimetics have a generally favorable safety profile, but gastrointestinal (GI) adverse effects are common [3, 4]. Furthermore, discontinuation of incretin agonists results in rapid BW regain, underscoring the need for long-term treatment with these peptide drugs [5, 6]. This highlights a role for small molecule drugs that either maintain BW loss following incretin agonist discontinuation or lower the rate and/or severity of adverse events when used in combination with them.

Long-chain acyl-coenzyme A (CoA) synthetases (ACSLs) attach a CoA group to long-chain fatty acids (LCFAs) as the first step in intracellular LCFA metabolism. Acyl-CoA fate is influenced by cell type and intracellular ACSL location. Because ACSLs appear to be highly compartmentalized, their Acyl-CoA products are likely, after synthesis, to enter a specific local intracellular pathway [7]. In humans, ACSL5 is expressed primarily in small intestine and colon, with less expression in liver and other tissues (https://gtexportal.org/home/ [GTEx Analysis Release V8 (dbGaP Accession phs000424.v8.p2)]). ACSL5 is most abundant in rodent small intestinal enterocytes responsible for LCFA absorption, re-esterification into triglycerides (TGs) and packaging of TGs into chylomicrons for systemic release [8-10]. ACSL5 is the primary ACSL expressed in mouse jejunal enterocytes, consistent with a 60% to 80% loss of jejunal total ACSL activity in *Acsl5* knockout (KO) mice [10, 11]. This suggests a compartmental model where ACSL5 directs jejunal LCFAs to TG re-esterification for systemic release, consistent with the delayed systemic appearance of dietary TGs in mice with global and intestine-specific KO of *Acsl5* [11, 12]. Recent work showed the favorable metabolic response to blocking this pathway; male mice with an intestine-specific *Acsl5* KO were protected from high-fat diet (HFD)-induced obesity, glucose intolerance, insulin resistance, and hepatic steatosis, and had lower circulating total cholesterol and increased circulating GLP-1 levels, in association with decreased food consumption (FC) and delayed gastric emptying (GE) [12].

Genes regulating body fat are shared with high fidelity by mice and humans [13, 14], and mouse KO phenotypes for known drug targets correlate well with drug efficacy [13, 15], indicating that mouse KO phenotyping may identify valuable anti-obesity drug targets. Our high-throughput phenotypic screen (HTPS) of mouse KO lines was designed to identify targets for drugs that could treat obesity and associated comorbidities [13, 16-18]. This HTPS, and confirmatory data from additional mouse cohorts, identified a favorable metabolic phenotype in both male and female *Acsl5* KO mice that included the above observations by Griffin et al [12] and extended them by showing minimal loss of lean body mass (LBM) along with ileal brake activation as a likely mechanism behind many of the metabolic effects of intestinal ACSL5 deficiency. This favorable metabolic phenotype led to a program for developing small molecule ACSL5 inhibitors (ACSL5i).

## Materials and Methods

### Knockout Mouse Models

**Figure 1.**
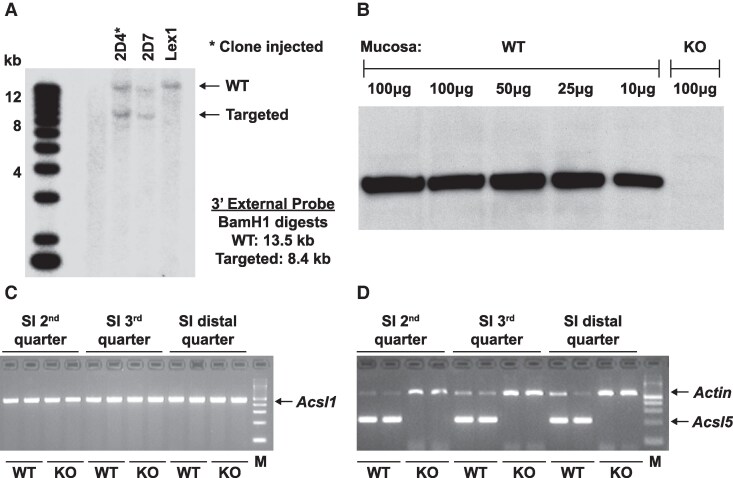
Studies confirming the *Acsl5* gene mutation. The targeting strategy used to disrupt the *Acsl5* locus is described in “Materials and Methods.” (A) Southern hybridization indicating proper gene targeting in the embryonic stem cell clones; clone 2D4 was selected for blastocyst injections. Lex1 represents untransfected embryonic stem cell DNA. (B) Western blot of jejunal mucosa from male *Acsl5* WT and KO mice probed with a goat polyclonal antibody raised against the 16 C-terminal amino acids of human ACSL5. Small intestinal *Acsl* mRNA transcripts were identified by PCR performed on mucosa from the distal three-quarters of the small intestine (SI) of male *Acsl5* KO and WT mice, using probes specific for mouse (C) *Acsl1* and (D) *Acsl5*. Abbreviations: Actin, β-Actin; M, molecular weight markers.

Our approach to KO and phenotype mouse orthologs of the estimated 5000 potential drug targets in the human genome (Genome 5000^TM^ project) is published [15-20]. Generation of *Acsl5* KO mice was initiated by constructing an *Acsl5* targeting vector using the Lambda KOS system [21]. The Lambda KOS phage library was screened by polymerase chain reaction (PCR) using exon 14–specific primers Acsl5-5 (5′-TTCGCCTCATGATCACTGG-3′) and Acsl5-4 (5′-TGAAGAAGTGAGTAGTGGGG-3′). PCR-positive phage super pools were plated and screened by filter hybridization using the 212 bp amplicon derived from primers Acsl5-5 and Acsl5-4 as probe. Genomic clone pKOS-31 was isolated and confirmed by sequence and restriction analysis. Gene-specific arms (5′-TCAAAGCTCTTCTTTGGTTTCCCAGGTGTTTGAAG-3′) and (5′-CAGTCCCATGGCAGAGAGCCTTACAAGTTAAGGAG-3′) were appended by PCR to a yeast selection cassette containing the URA3 marker. The yeast selection cassette and pKOS-31 were co-transformed into yeast, and clones that had undergone homologous recombination to replace a 744-bp region containing exons 15-17 with the yeast selection cassette were isolated. The yeast cassette was then replaced with a LacZ/Neo selection cassette to complete the *Acsl5* targeting vector. The Not I linearized targeting vector was electroporated into 129/SvEv^Brd^ (Lex-1) ES cells. G418/FIAU resistant ES cell clones were isolated, and correctly targeted clones were identified and confirmed by Southern analysis using a 351-bp 5′ external probe (44/24), generated by PCR using primers Acsl5-44 (5′-TCCGTTATCGAAGTTGTTTCC-3′) and Acsl5-24 (5′-TTGGCTTCATTTTGTACCTGG-3′), and a 551-bp 3′ external probe (41/43), amplified by PCR using primers Acsl5-41 (5′-GCAAGTTTTTGTCCATGGGG-3′) and Acsl5-43 (5′-ATGAGGGAAGTGAATCTGGG-3′). Southern analysis using probe 44/24 detected a 13.5-kb wild-type (WT) band and 4.5-kb mutant band in Bam HI digested genomic DNA (data not shown) while probe 41/43 detected a 13.5-kb WT band and 8.4-kb mutant band in Bam HI digested genomic DNA (Fig. 1A). Two targeted ES cell clones were identified and microinjected into C57BL/6J-Tyr*^c−Brd^* (albino) blastocysts to generate chimeric animals which were bred to C57BL/6J-Tyr*^c−Brd^* (albino) females; the resulting heterozygous (HET) offspring were interbred to produce homozygous *Acsl5*-deficient (KO) mice. All *Acsl5* KO and WT mice reported here were maintained on a C57BL/6J-Tyr*^c−Brd^* X 129SvEvBrd hybrid background. Mouse genotyping at the *Acsl5* locus was performed by screening tail biopsy DNA samples using quantitative PCR for the *Neo* cassette, which enabled discrimination of 0, 1, or 2 gene disruptions representing Acsl5 WT, HET, and KO mice, respectively.

### Mouse Care and Study

All mouse studies were performed in strict accordance with recommendations in the Guide for the Care and Use of Laboratory Animals of the National Institutes of Health [22]. The protocols for all studies were approved by the Lexicon Institutional Animal Care and Use Committee (OLAW Assurance Number, A4152-01; AAALAC International Accreditation Number, 001025). General methods for mouse care have been published [18]. Briefly, mice were housed together, with a maximum of 5 mice/cage, in a temperature-controlled (24 °C) environment on a fixed 12-h light/12-h dark cycle and with free access to food and water. Mice were fed either a standard rodent chow containing 22% kcal from fat (9F 5020; Purina, St Louis, MO, USA), a purified 3.85 kcal/g low-fat diet (LFD) containing 10% kcal from fat (D12450B, Research Diets, New Brunswick, NJ, USA), a purified 4.73 kcal/g high-fat diet (45% HFD) containing 45% kcal from fat (D12451; Research Diets), a purified 5.24 kcal/g high-fat diet (60% HFD) containing 60% kcal from fat (D12492; Research Diets), or a 4.4 kcal/g high-fat diet (Clinton 40% HFD) containing 40% kcal from fat (D12107B; Research Diets). Unless stated otherwise, all studies were performed on adult mice.

### Primary High-Throughput Phenotypic Screen

As part of Lexicon's Genome5000^TM^ program to KO and phenotype the druggable mouse genome, adult WT and KO mice were evaluated by a series of HTPS assays [16, 17, 19]. Among dozens of assays included in the HTPS protocol were assays evaluating body composition and serum chemistries performed on a cohort of mice fed chow diet from weaning; this cohort routinely consisted of 4 WT and 8 KO mice, half male and half female. Body composition was analyzed on these chow-fed mice by dual-energy x-ray absorptiometry (DXA) using a GE/Lunar PIXImus scanner (GE Medical Systems, Madison, WI) [13, 18, 23]. For the *Acsl5* KO line, mean KO body fat/mean WT littermate body fat was calculated for both male and female mice, and then these male and female values were averaged and multiplied × 100, yielding a normalized body fat value; normalized values for other HTPS assays, and for body composition measurements on additional cohorts, were also obtained using this approach. Serum chemistries were assayed on retro-orbital blood obtained in the fed state from conscious, unanesthetized male and female *Acsl5* KO and WT mice by Cobas Integra 400 analyzer (Roche Diagnostics, Indianapolis, IN) [16, 22, 24, 25].

### Body Composition Measurements on Additional Cohorts

Body composition was measured on all additional male and female mouse cohorts by quantitative magnetic resonance (QMR; Echo Medical Systems, Houston, TX, USA) as described [18, 23].

### Oral Glucose Tolerance Tests

Oral glucose tolerance tests (OGTTs) were performed on conscious, unanesthetized male and female KO and WT mice as described previously [26]. After an overnight fast, mice were bled from the retro-orbital plexus at baseline and then received 2 g/kg glucose by oral gavage. Whole-blood samples obtained from the retro-orbital plexus at 0, 30, and 60 minutes were directly assayed for glucose levels by ACCU-CHEK Aviva glucometer (Roche, Indianapolis, IN, USA); serum obtained at 0 and 30 minutes was used to measure insulin levels (Ultra-Sensitive Rat Insulin ELISA Kit, Cat. 90060; Crystal Chem, Downers Grove, IL, USA).The homeostatic model assessment (HOMA) insulin sensitivity index (ISI) and the composite ISI were measured using OGTT glucose and insulin data for each mouse [22, 27, 28].

### Tyloxapol Gastric Lipid Loading Bioassay

After an overnight fast, male and female mice received 100 µL of tyloxapol (Triton WR1339, catalog #T-8761; Sigma-Aldrich, St. Louis, MO) as a 20% solution in 0.9% NaCl, by tail vein injection, followed by a 10 µL/g oral gavage bolus of olive oil containing ^3^H triolein (glycerol tri [9,10 (n)-^3^H] oleate, American Radiolabeled Chemicals, St. Louis, MO), where ^3^H triolein (1 µCi/µL) was mixed with olive oil at a ratio of 1 µL (1 mCi) ^3^H triolein to 299 µL olive oil. For the tyloxapol gastric lipid loading (TGLL) bioassay, blood samples were drawn at 2, 4, and 6 hours and ^3^H was quantified by mixing 50 µL serum with 20 mL scintillation fluid and measuring ^3^H disintegrations per minute (DPMs) using a Beckman Coulter LS6500 liquid scintillation counter (Beckman Coulter Inc., Irving, TX).

### Small Molecule ACSL5i

Discovery of small molecule ACSL5i began with high-throughput screening of a library of ∼300 000 compounds using a radiometric enzyme assay. Several classes of hits were identified. Hit compounds were synthesized and optimized at Lexicon Pharmaceuticals. ^1^H NMR spectra were collected on Bruker ARX300, DRX400, or DPX400 (Bruker Corp., Billerica, MA). Analytical HPLC spectra were collected on a Shimadzu HPLC system equipped with an auto-sampler and a UV detector, 220 and 254 nm (Shimadzu Scientific Instruments, Columbia, MD). Mass spectra were obtained on Waters ZQ or ZMD LCMS system equipped with an auto-sampler, an ELSD detector, a UV detector (220 and 254 nm) and a Mass detector (Waters Corp. Milford, MA). The purity of all compounds tested exceeded 98%. For all in vivo studies, the vehicle for ACSL5i delivery was 10% solutol.

### ACSL Enzyme-Coupled Assay

To generate ACSL proteins, His-6 C-terminally tagged human *ACSL1* (*hACSL1*; accession AAB00959), *hACSL3* (accession EAW70809), *hACSL5* (accession AB033899), mouse *Acsl1* (m*Acsl1;* accession AAH56644), and m*Acsl5* (accession AAH31544) were cloned into the Invitrogen pFastBac1 expression vector (Thermo Fisher Scientific, Waltham, MA; catalog #10360-014) and transfected into Sf9 insect cells for expression in Gibco SF-900 II serum free medium (Thermo Fisher Scientific, catalog #10902088) as recommended by the manufacturer (Invitrogen, Carlsbad, CA). After cell paste from a 4-liter culture was resuspended in 100 mL Lysis Buffer (50 mM Tris-HCl pH 8, 500 mM NaCl, 1 mM DTT, 10% glycerol, 1% Triton X-100, 1 mM PMSF, Roche cOmplete EDTA-free Protease Inhibitor Cocktail [Roche, Indianapolis, IN]), sonicated, and centrifuged, the supernatant was loaded onto 5 mL Ni-NTA resin pre-equilibrated with Lysis Buffer. Following washes with 100 mL Lysis Buffer and then with 100 mL Lysis Buffer without Triton X-100, ACSL was eluted with 15 mL Eluting Buffer (50 mM Tris-HCL pH 8, 500 mM NaCl, 1 mM DTT, 10% glycerol, 500 mM Imidazole, 3 mM MgCl_2_, 10 mM ATP). Active fractions were dialyzed against 50 mM TRIS-HCL pH 8, 50% glycerol, 150 mM NaCl, 1 mM EDTA, 0.5 mM TCEP, aliquoted and stored at −80 °C.

The ACSL assay is an enzyme-coupled fluorescence method where the ACSL reaction is coupled with Acyl-CoA Oxidase and Peroxidase reactions. In the final step, H_2_O_2_ reacts with the Amplex Red reagent to produce a fluorescent product (Resorufin).


$$\mathrm{FFA}+\mathrm{CoA}+\mathrm{ATP}\;\underset{\to }{\mathrm{ACSL}}\;\mathrm{Acyl}\text{-}\mathrm{CoA}+\mathrm{AMP}+PP_{i}$$

$$\begin{array}{rl} \mathrm{Acyl}\text{-}\mathrm{CoA}+O_{2}+\mathrm{ATP}\;\underset{\to }{\mathrm{Acyl}-\mathrm{CoA}\;\mathrm{Oxidase}} & \mathrm{Trans}\text{-}2\text{-}\mathrm{enoyl}\text{-}\mathrm{CoA}\\ & +H_{2}O_{2} \end{array}$$

$$\begin{array}{rl} H_{2}O_{2}\;+\;\mathrm{Amplex}\;\mathrm{Red}\;\mathrm{reagent}\;\underset{\to }{\mathrm{Peroxidase}} & \mathrm{Resorufin}\;\\ & \left(\mathrm{fluorescence}\right) \end{array}$$

Reaction buffer consisted of 150 mM MOPS pH 7.6, 12 mM MgCl2, 0.55 mM Triton X100, 0.05 U/mL Acyl-CoA Oxidase (catalog #T17, Asahi Kasei Corporation, Tokyo, Japan), 0.01 U/mL Horseradish Peroxidase (catalog #P8375, Sigma-Aldrich), 0.05 mM Amplex Red (catalog #A22177, Invitrogen), 30 µM Na Oleate (catalog #O7501, Sigma-Aldrich), 30 µM CoA (catalog #234101, Calbiochem, San Diego, CA) and 1 mM ATP (catalog #A2383, Sigma-Aldrich). Reagents were premixed and combined in 384-well plates (catalog #781076, Greiner Bio-One, Kremsmunster, Austria) as follows: 1) 10 µL buffer; 2) compound in DMSO (202.5 nL, final 0.675% DMSO); 3) 20 µL Enzyme Mix (Acyl-CoA Synthetase, Acyl-CoA Oxidase, Peroxidase); and 4) 20 µL Substrate Mix (Oleate, Co-A, ATP, Amplex Red). After addition and mixing, plates were read continuously (excitation 535, emission 590) on an Envision reader (PerkinElmer, Waltham, MA). The amount of ACSL added allowed a linear reaction for between 10 and 60 minutes.

### Thin Layer Chromatography

After chow-fed male and female mice were acclimatized to individual housing, ^3^H triolein was gavaged as in the TGLL assay. All feces were collected over the next 24 hours, mixed with 3 mL PBS, then extracted with 20 mL chloroform/methanol (2:1) and 2 mL 5% H_2_SO_4_. After centrifugation, 5 µL of organic layer from each sample was loaded in a thin layer chromatography (TLC) apparatus containing a silica gel 20 × 20 cm TLC plate (Whatman International Ltd, Maidstone, UK, Cat. No. 4865-821); a Whatman 1 filter paper served as wick. Also loaded was a 5 µL standard from a mixture of 20 µL/mL glycerol oleate (TG), 20 µL/mL of oleic acid (free fatty acid [FFA]) and 20 µL/mL glycerol dioleate (diacylglycerol [DAG]) in 1 mL of chloroform/methanol (2:1). After sample separation for 1 hour using a solvent of n-heptane/diethyl ether/acetic acid (82:18:1), the plate was dried for 10 minutes, and an image was obtained after a 30-minute staining in iodine. For each sample, silica from individual bands was scraped with a razor blade into a vial, crushed into a fine powder with a wooden tip and solubilized with 500 µL chloroform/methanol (2:1); then, 10 µL were mixed with 20 mL scintillation fluid and vials were read for DPM as described for the TGLL assay. A similar TLC protocol was followed for adult male WT mice receiving either vehicle (n = 5) or 100 µg/g orlistat (n = 5) in the olive oil gavage except that ^3^H triolein was not added to the olive oil.

### Gastric Emptying

In the morning, nonfasted male mice were dosed with tyloxapol by tail vein and ^3^H triolein in olive oil by gavage as in the TGLL assay. After dosing, mice were individually caged on wire grids over paper towels for feces collection. After 6 hours, mice were bled and stomach, small intestine, cecum, and large intestine were collected and snap-frozen in 4 different tubes. Each of these organs, and feces, were extracted in the same way using the Dole method [29]. After extraction, 500 μL of each extract and 50 μL serum were added to 18 mL Hionic Fluor (PerkinElmer) and DPMs measured as in the TGLL assay.

### Active GLP-1 Studies

The effects of *Acsl5* KO, LP-856866 and/or sitagliptin on circulating active GLP-1 (aGLP-1) levels over time were assessed after baseline delivery of a 45% HFD meal. The HFD meal was prepared by adding 50 g of 45% HFD powder to 70 mL boiling milliQ water for 10 minutes and then mixing with a hand mixer in a 500 mL beaker which was kept in warm water while dosing. Chow-fed male and female mice were fasted overnight, pretreated with either vehicle (10% solutol) or compounds by oral gavage, and then challenged with 15 µL/g BW of the HFD meal by oral gavage 30 minutes later. Blood samples for aGLP-1 measurement were obtained by retro-orbital bleeding of unanesthetized mice at multiple timepoints up to 240 minutes after administration of the HFD meal. After the immediate transfer of each blood sample into an ice-cold EDTA-containing tube, Dipeptidyl peptidase (DPP) IV inhibitor solution provided in the aGLP-1 ELISA kit was immediately added to the tube in a ratio of 10 μL of DPP IV inhibitor solution/mL blood. The sample was mixed and centrifuged immediately at 1000*g* for 10 minutes at 4 °C, followed by collection of plasma for aGLP-1 assay by ELISA (Millipore Cat# EGLP-35K, RRID:AB_2737305, Linco Research, St. Charles, MO) as described previously [30, 31].

### Blood Analytes

Unless stated otherwise, kits were used to measure total cholesterol (catalog number 439-17501, Wako Diagnostics, Richmond, VA) and TG (L-type TG-M kit, Wako Diagnostics) on male and female mice.

### Liver Fat Studies

Liver TGs extracted from male and female mice were quantitated as described [23]. Liver tissue was prepared and stained with hematoxylin and eosin as described [23], masked for treatment group, and then scored for fat content by the pathologist using the following key: absent = 0, minimal = 1, mild = 2, moderate = 3, marked = 4, severe = 5.

### Western Blotting

Male *Acsl5* KO and WT mice were fasted overnight prior to small intestine harvesting. While on ice, the middle third of the small intestine was flushed with ice-cold 0.9% NaCl and then cut open end to end. The mucosa was gently scraped off with a glass slide, weighed, homogenized in 10× volume of 50 mM Tris pH 7.4, 150 mM NaCl, 2mM EDTA, 1.0% NP-4, 0.1% SDS + protease inhibitors, and centrifuged at 12 500 rpm for 15 minutes at 4 °C. After supernatants were assayed for BCA total protein, WT and KO mucosal samples were loaded onto a reduced 8% Tris-Glycine gel and separated at 4 °C at 80 V. Proteins were transferred to a PVDF membrane and probed overnight at 4 °C with a 1:200 dilution of goat polyclonal antibody raised against the 16 C-terminal amino acids of hACSL5 (Cat# sc-47999, RRID:AB_ 2305124, Santa Cruz Biotechnology, Inc., Dallas, TX); a 1:2000 dilution of donkey anti-goat IgG-HRP (Cat# sc-2020, RRID:AB_631728, Santa Cruz Biotechnology, Inc.) served as secondary antibody.

### Small Intestinal Acsl Transcripts

Mucosa samples from the distal three-quarters of the small intestines of 2 male *Acsl5* KO mice and 2 male WT littermates were collected as described above for the Western blotting method. Total RNA was extracted from intestinal mucosa samples using a bead homogenizer (BioSpec Products, Inc., Bartlesville, OK) and Trizol reagent (Invitrogen) as recommended by the manufacturer. Reverse transcription was performed to produce cDNA using 5 mg total RNA and SuperScript II (Invitrogen) along with random hexamer primers, again as recommended by the manufacturer. PCR amplification was performed for all samples on the same day, using 1 mL of each reverse transcription product in an initial denaturing step of 95 °C for 2.5 minutes followed by 40 cycles of 95 °C (30 seconds), 59 °C (20 seconds), and 70 °C (1 minute) in the presence of oligonucleotides complementary to *Acsl1* (forward or F primer: GCTGATTGACATTCGGCAGTACGTG; reverse or R primer: GCGGGACGACCACCATTGAGTAAGA) or *Acsl5* (F: AGTCTTGACATTCTTCAGGGCTGCAATGGG; R: TGATGCAGATCTCGCCTTCGTTGTT). Mouse *β-actin* (GenBank accession number M12481) served as an internal control for sample handling, using complementary oligonucleotides F: GGCTGGCCGGGACCTGACGGACTACCTCAT and R: GCCTAGAAGCACTTGCGGTGCACGATGGAG. PCR products were separated on a 2.5% agarose gel along with a 100-bp ladder.

### Statistics

Data are presented as mean ± SD. Unless stated otherwise, comparisons between 2 groups were analyzed by unpaired Student's *t* test or, if data variances were significantly different between the 2 groups by F test, data were analyzed by the nonparametric Mann–Whitney test; comparisons among 3 or more groups were analyzed by one-way ANOVA followed by Dunnett's multiple comparisons test or, if SDs among the groups were significantly different by either the Brown-Forsythe or Bartlett's test, data were analyzed by the nonparametric Kruskal-Wallis test followed by Dunn's multiple comparisons test. All statistical tests were performed using PRISM 4.03 (GraphPad Software, Inc., La Jolla, CA, USA). Differences were considered statistically significant when *P* < .05.

## Results

### Acsl5 KO Mice Are Healthy and Have Acsl1 but Not Acsl5 Transcripts in the Distal Small Intestine

*Acsl5* KO mice were generated using the strategy outlined in “Materials and Methods.” KO mice were viable, healthy, and exhibited a normal Mendelian ratio of 2705 WT, 5327 HET, and 2683 KO mice. Southern blotting suggested successful deletion of *Acsl5* exons 15-17 (Fig. 1A), consistent with the presence of ACSL5 protein in small intestinal mucosa of WT mice but not *Acsl5* KO mice (Fig. 1B). PCR analysis confirmed the presence of *Acsl1*, but not *Acsl5*, transcripts in the distal small intestine of *Acsl5* KO mice (Fig. 1C and 1D).

### HTPS Demonstrated a Metabolic Phenotype in Acsl5 KO Mice

When chow-fed *Acsl5* KO and WT mice underwent HTPS, the *Acsl5* KO mice showed trends toward lower serum TGs (55% lower), serum total cholesterol (24% lower), blood glucose (19% lower), and body fat (19% lower), along with conserved LBM, when compared to WT littermates. These results suggested the need to study additional cohorts for the presence of a favorable metabolic phenotype.

### HFD-Fed Acsl5 KO Mice Are Thinner Than WT Mice but Maintain Their LBM

**Figure 2.**
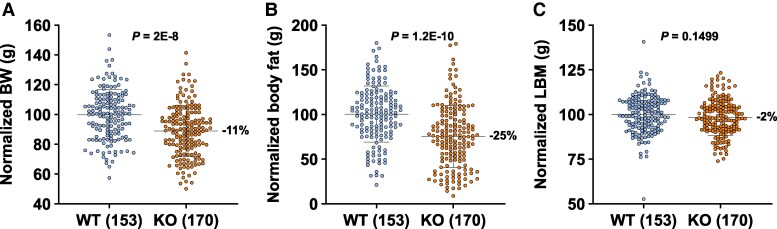
Body composition data of HFD-fed *Acsl5* KO and WT mice. Body composition was measured by quantitative magnetic resonance (QMR) in 5 cohorts of male and female *Acsl5* KO and WT mice fed HFD for at least 20 weeks (Table 1C). For each cohort of male or female mice, the mean WT value was normalized to 100% for each parameter measured, and the data were then pooled for the 153 WT and 170 KO HFD-fed mice studied: (A) Body weight (BW); (B) Body fat in grams; and (C) Lean body mass (LBM) in grams.

Our initial focus was on whether *Acsl5* KO mice were thin. Early studies showed that these KO mice were not thin at weaning (Table 1A). In contrast, multiple cohorts of male and female *Acsl5* KO mice fed chow diet from weaning showed a trend toward lower body fat, with BW and LBM that were not different from WT (Table 1B); when normalized body fat data were pooled from all male and female chow-fed cohorts, KO mice showed a modest but significant 10% decrease (WT: 100% ± 47% [n = 144] vs KO: 90% ± 40% [n = 138], *P* < .05), confirming HTPS data. *Acsl5* KO mice showed a more exaggerated thin phenotype when additional mouse cohorts were fed 45% HFD for at least 20 weeks (Table 1C). As shown in Fig. 2A-2C, HFD-fed *Acsl5* KO mice had lower BW and body fat, but maintained their LBM, when all mouse data were pooled. Both sexes were comparably thinner, showing identical 25% decreases in body fat (female WT: 100% ± 38% [n = 83] vs KO: 75% ± 38% [n = 87], *P* < .001; male WT: 100% ± 22% [n = 70] vs KO: 75% ± 32% [n = 83], *P* < .001) and 18% decreases in % body fat (female WT: 100% ± 23% vs KO: 82% ± 28%, *P* < .001; male WT: 100% ± 14% vs KO: 82% ± 25%, *P* < .001). These findings prompted further study of HFD-fed *Acsl5* KO mice.

**Table 1.** Body composition of Acsl5 KO and WT mice

Cohort	Age	Sex	Genotype	N	BW (g)	Body fat (g)	Body fat (%)	LBM (g)
**A. *Acsl5* KO and WT mice at weaning**								
1	5 wks	Female	WT	18	18 ± 1	2.5 ± 0.5	14 ± 2	12 ± 1
			KO	15	17 ± 2	2.6 ± 0.5	15 ± 2	12 ± 1
		Male	WT	9	23 ± 2	2.5 ± 0.4	11 ± 2	16 ± 2
			KO	18	22 ± 2	2.8 ± 0.5	13 ± 2**	15 ± 2
2	5 wks	Female	WT	11	18 ± 1	2.4 ± 0.3	14 ± 1	13 ± 1
			KO	6	18 ± 1	2.5 ± 0.2	14 ± 2	13 ± 1
		Male	WT	18	23 ± 2	2.9 ± 0.5	12 ± 2	17 ± 2
			KO	12	22 ± 3	2.8 ± 0.3	13 ± 1	16 ± 2
**B. *Acsl5* KO and WT mice fed chow diet from weaning**								
3	15 wks	Female	WT	13	26 ± 5	5.5 ± 3.5	20 ± 8	20 ± 2
			KO	14	26 ± 2	4.3 ± 1.2	17 ± 4	21 ± 2
4	18 wks	Female	WT	14	27 ± 4	6.1 ± 3.0	22 ± 7	21 ± 2
			KO	14	27 ± 4	5.4 ± 2.3	19 ± 6	22 ± 2
		Male	WT	9	34 ± 2	4.2 ± 1.1	12 ± 3	30 ± 1
			KO	14	33 ± 3	3.6 ± 1.0	11 ± 3	29 ± 3
5	26 wks	Female	WT	12	31 ± 7	9.5 ± 4.8	29 ± 9	21 ± 2
			KO	13	28 ± 4	7.0 ± 3.0	24 ± 7	21 ± 2
6	18 wks	Female	WT	13	31 ± 7	8.4 ± 4.7	25 ± 9	23 ± 3
			KO	11	29 ± 7	7.2 ± 5.5	23 ± 11	22 ± 2
		Male	WT	17	37 ± 5	6.7 ± 3.5	17 ± 7	30 ± 3
			KO	15	38 ± 4	6.6 ± 2.7	17 ± 6	31 ± 3
7	18 wks	Female	WT	30	25 ± 3	5.5 ± 2.1	21 ± 5	20 ± 2
			KO	25	26 ± 3	5.1 ± 2.2	19 ± 7	21 ± 2*
		Male	WT	36	35 ± 5	6.3 ± 2.9	17 ± 6	29 ± 2
			KO	32	34 ± 4	6.1 ± 2.7	18 ± 7	28 ± 3
**C. *Acsl5* KO and WT mice fed 45% HFD for at least 20 weeks**								
3	36 wks*^a^*	Female	WT	13	41 ± 7	18.0 ± 5.3	43 ± 9	23 ± 4
			KO	13	36 ± 7	11.5 ± 5.8**	30 ± 10**	25 ± 2
	28 wks*^a^*	Male	WT	8	45 ± 8	16.5 ± 4.6	36 ± 4	28 ± 4
			KO	9	42 ± 4	13.3 ± 3.6	31 ± 6*	29 ± 1
4	39 wks*^b^*	Female	WT	14	45 ± 10	20.4 ± 7.3	44 ± 7	25 ± 3
			KO	14	37 ± 8*	13.6 ± 6.5*	35 ± 10*	24 ± 2
		Male	WT	8	55 ± 4	19.2 ± 2.4	35 ± 4	36 ± 3
			KO	14	46 ± 8**	13.9 ± 5.8*,†	29 ± 9	32 ± 3*
8	25 wks*^b^*	Female	WT	12	27 ± 4	6.4 ± 2.4	23 ± 6	21 ± 2
			KO	9	27 ± 2	6.5 ± 2.8	23 ± 8	21 ± 1
		Male	WT	8	39 ± 6	12.5 ± 4.5	31 ± 7	27 ± 2
			KO	13	36 ± 7	8.4 ± 4.6	22 ± 8*	28 ± 3
9	38 wks*^c^*	Female	WT	29	42 ± 9	17.6 ± 7.3	40 ± 10	24 ± 3
			KO	31	38 ± 8	13.7 ± 6.9*	34 ± 10*	24 ± 2
		Male	WT	28	56 ± 5	22.1 ± 3.4	39 ± 4	34 ± 3
			KO	30	48 ± 11***,†	15.2 ± 7.4***,†	30 ± 11***,†	32 ± 4
10	34 wks*^d^*	Female	WT	15	37 ± 8	14.3 ± 6.6	36 ± 10	23 ± 2
			KO	20	33 ± 7	10.2 ± 5.5	29 ± 11	23 ± 2
		Male	WT	18	51 ± 6	17.7 ± 4.6	35 ± 7	33 ± 3
			KO	17	47 ± 7	16.3 ± 4.6	34 ± 6	31 ± 3

Abbreviations: N, number of mice; BW, body weight; g, grams; LBM, lean body mass; wks, weeks; WT, wild type; KO, knockout. Mice were maintained on HFD for:

^a^21 weeks;

^b^20 weeks;

^c^29 weeks;

^d^25 weeks. KO mice different from WT mice,

^*^ *P* < .05;

^**^ *P* < .01;

***P < .001;

^*†*^statistical analysis by Mann-Whitney test.

### Decreased Intestinal TG Uptake During 6 Hours After Oral Delivery to Acsl5 KO Mice

**Figure 3.**
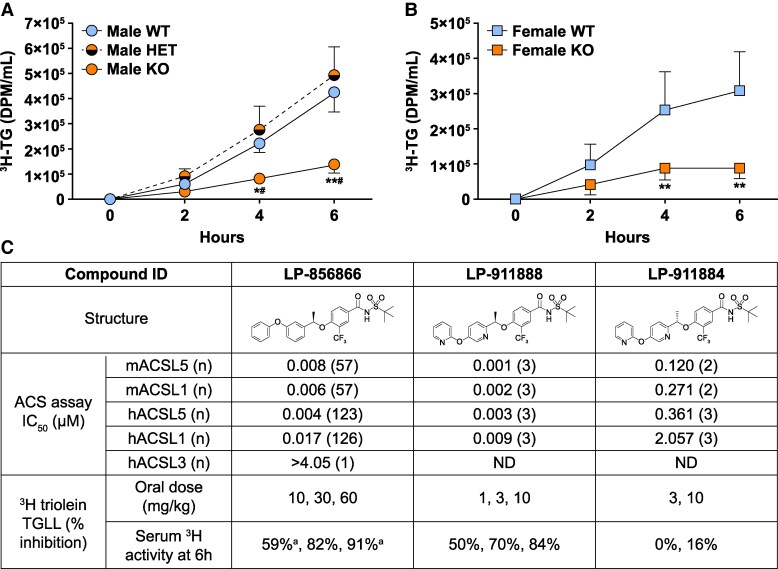
^3^H triolein TGLL bioassay in *Acsl5* KO mice, and small molecule ACSL5i compounds identified by ACS and ^3^H triolein TGLL assays. (A) ^3^H triolein TGLL bioassay performed in male KO mice. After an overnight fast, mice (11 WT, 10 HET, 7 KO) received tyloxapol followed by a bolus of olive oil containing ^3^H triolein by oral gavage; ^3^H disintegrations/minute (DPM) were then measured in serum samples at 2, 4, and 6 hours. (B) ^3^H triolein TGLL bioassay performed in female KO mice. After an overnight fast, mice (29 WT, 19 KO) underwent a TGLL bioassay study as described in (A) above. KO mice different from WT mice: *P < .05; **P < .01. ^#^Statistical analysis by Kruskal-Wallis test followed by Dunn's multiple comparisons test. (C) ACSL5i structure and activity in ACS and ^3^H triolein TGLL assays. Top panels: compound structures. Middle panels: acyl-coenzyme A synthetase (ACS) assay IC_50_ values; Abbreviations: hACSL5, human ACSL5; hACSL1, human ACSL1; hACSL3, human ACSL3; mACSL5, mouse ACSL5; mACSL1, mouse ACSL1; ND, not determined; (n), number of times assayed. Bottom panels: ^3^H triolein TGLL bioassay dose and % inhibition data; 6 h, 6 hours; ^a^all data from the same study.

Because ACSL5 has been linked to re-esterification of TGs in small intestinal enterocytes, we next studied the postprandial response of *Acsl5* KO and WT mice to an acute oral load of olive oil containing ^3^H triolein. These gastric lipid loading challenges were performed in the presence of tyloxapol (TGLL) to block peripheral lipolysis; since ^3^H triolein was delivered orally, ^3^H appearance in the circulation over the next 6 hours reflected intestinal TG absorption. As shown in Fig. 3, systemic TG appearance was blunted in *Acsl5* KO mice undergoing ^3^H triolein TGLL challenges; male and female KO mice had 67% and 71% less circulating ^3^H triolein, respectively, after 6 hours (Fig. 3A and 3B), while male HET and WT mice had a comparable response (Fig. 3A). The robustness of the response in *Acsl5* KO mice suggested that the ^3^H triolein TGLL challenge might be a useful in vivo bioassay to identify small molecule ACSL5i.

### Identification of ACSL5i and a Less-Active Enantiomer

ACSL5i were first identified by small molecule library screening followed by screening hits in vitro with acyl-CoA synthetase (ACS) assays for mACSL5, mACSL1, hACSL5, and hACSL1, followed by in vivo screening using the ^3^H triolein TGLL bioassay. Structures and assay results for the ACSL5i LP-856866 and LP-911888 are presented in Fig. 3C, along with the LP-911888 enantiomer LP-911884; enantiomers have identical chemical structure but opposite chirality. LP-911888 and LP-856866 were potent ACSL5i in the ACS screen, while the LP-911888 enantiomer LP-911884 was much less active. LP-856866 and LP-911888 were also potent inhibitors of the most closely related family member, ACSL1. LP-856866 was highly selective against ACSL3. At the doses employed, LP-856866 and LP-911888, but not LP-911884, inhibited intestinal TG uptake in the ^3^H triolein TGLL bioassay comparably to the inhibition observed in *Acsl5* KO mice.

### ACSL5i Reproduce the Thin Phenotype of HFD-Fed Acsl5 KO Mice

**Figure 4.**
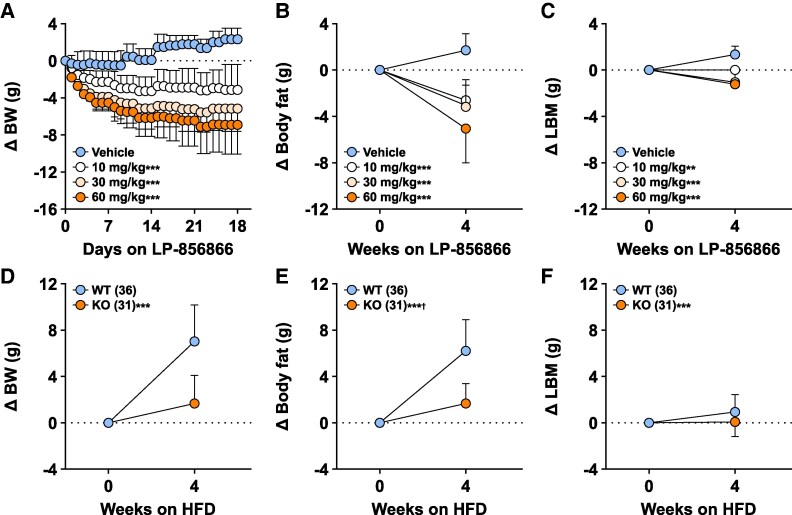
Body composition changes by QMR of DIO mice treated with the ACSL5i LP-856866 compared to *Acsl5* KO mice switched from chow to HFD. Effects of 4-week treatment with either vehicle or LP-856866 delivered by dietary admixture on (A) body weight (BW); (B) body fat; and (C) lean body mass (LBM) of male DIO mice maintained on 45% HFD from weaning (n = 8/group). Effects of switching male *Acsl5* KO and WT mice from chow to 45% HFD for 4 weeks on (D) body weight; (E) body fat; and (F) LBM. Dotted line identifies 0 change at all time points. Change from baseline to 4-week values of KO mice or LP-856866-treated mice different from WT or vehicle-treated mice: *P < .05; **P < .01; ***P < .001. ^†^Statistical analysis by Mann–Whitney test.

When mice with diet-induced obesity (DIO; 45% HFD intake for more than 16 weeks) were treated with LP-856866, they lost BW and body fat in a dose-dependent manner, with less LBM loss (Fig. 4A-4C). The differences in BW and body fat, and the sparing of LBM, that developed between LP-856866- and vehicle-treated mice were reproduced when chow-fed *Acsl5* KO and WT mice were switched to 45% HFD for 1 month (Fig. 4D-4F). We next sought to understand the mechanism behind development of this thin phenotype.

### Decreased HFD Consumption by ACSL5-Deficient Mice

**Figure 5.**
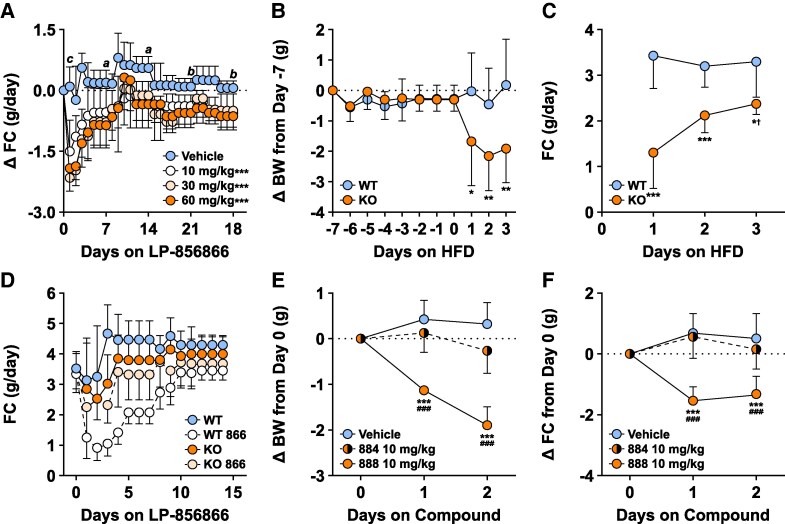
Effects of ACSL5 deficiency on food consumption and body weight. (A) Change from baseline in daily food consumption (FC) of the DIO mice from Fig. 3A during the 28 days of LP-856866 (866) treatment; FC of vehicle-treated group different from each 866-treated group on individual days: *a*, *P* < .05; *b*, *P* < .01; *c*, *P* < .001. Area under the curve of 28-day FC different from WT, ***P < .001. After male *Acsl5* KO (n = 9) and WT (n = 8) mice were acclimated to individual cages for 1 week, their BW was measured on Day -7; these mice were then switched from chow to 45% HFD on Day 1 and followed for (B) change in body weight (BW) from Day -7 value and (C) daily FC. KO mice different from WT mice: *P < .05; **P < .01; ***P < .001. ^†^Statistical analysis by Mann–Whitney test. (D) Effect of LP-856866 or vehicle on FC of male *Acsl5* KO mice (5/group) and WT mice (10/group) maintained on 45% HFD for 32 weeks and individually housed for 2 weeks. Total FC over day 1-10: WT different from WT + LP-856866, *P* < .001 by Mann-Whitney test; KO not different from KO + LP-856866. Effect of LP-911888 (888) and its less active enantiomer LP-911884 (884) on change from Day 0 for (E) BW and (F) FC of male mice maintained on 45% HFD from weaning and individually housed for 2 weeks prior to study; n = 8 mice/group. Dotted line identifies 0 change at all time points. 888 treated mice different from vehicle: ^###^*P* < .001; 888 treated mice different from 884 treated mice: ***P < .001.

Initiating LP-856866 treatment in DIO mice was associated with decreased HFD intake that was less marked with time but continued for 28 days (Fig. 5A). Because switching *Acsl5* KO and WT mice to 45% HFD led to similar rapid differences in BW and body fat, we tested if this was also due to lower FC. Chow-fed *Acsl5* KO and WT mice had stable BW from Day −7 to Day 0, but their switch to HFD on day 1 coincided with a rapid decrease in BW and FC of *Acsl5* KO mice (Fig. 5B and 5C). Importantly, in mice maintained on 45% HFD, LP-856866 lowered FC in WT but not in *Acsl5* KO mice (Fig. 5D), suggesting that LP-856866 lowered FC due to on-target inhibition of ACSL5 and not to off-target effects through ACSL1 or other pathways. The ability of the ACSL5i LP911888, but not its less-active enantiomer LP-911884, to inhibit FC (Fig. 5E) and BW (Fig. 5F) in HFD-fed WT mice provides further evidence that ACSL5 inhibition drives the hypophagia, because LP-911884 should minimally inhibit ACSL5 at the dose provided in this study.

### Mice With ACSL5 Deficiency Prefer LFD Over HFD

**Figure 6.**
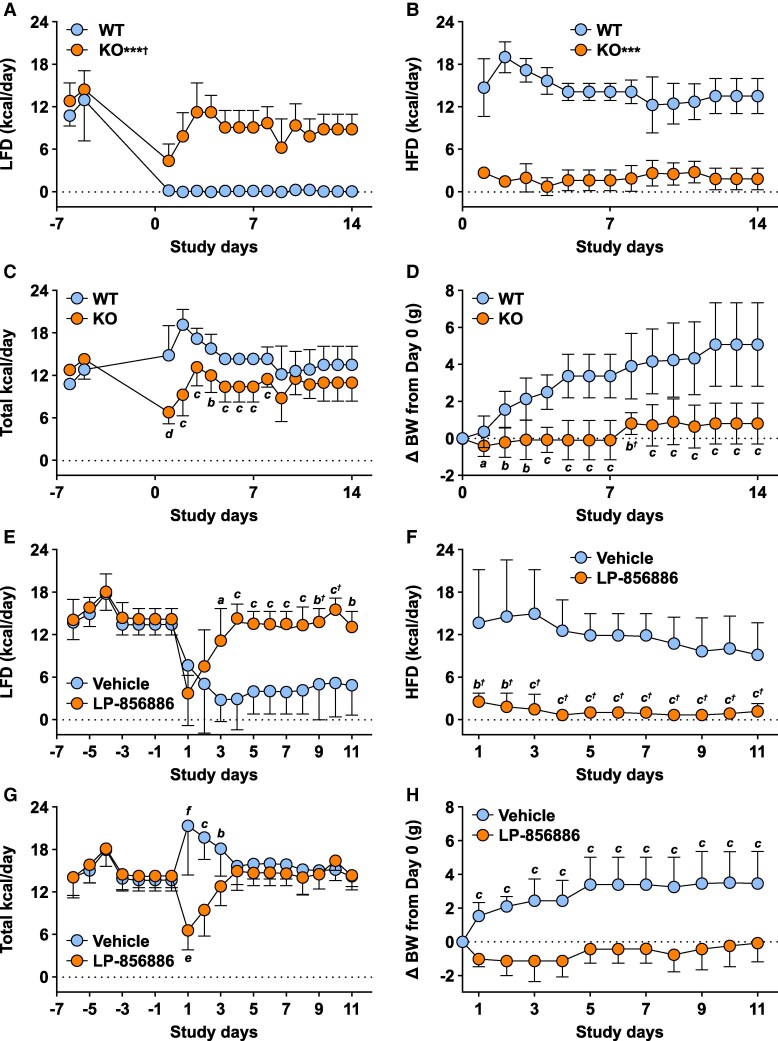
Effects of ACSL5 deficiency on fat preference. Chow-fed male *Acsl5* KO (n = 9) and WT (n = 8) mice were individually housed and acclimatized on 10% LFD for 1 week and then provided simultaneously with 60% HFD and 10% LFD starting on Day 1: (A) daily intake of 10% LFD; (B) daily intake of 60% HFD; (C) daily total kcal intake; (D) change in BW from Day 0 value. For panels (A) and (B), KO total Day 1 to Day 14 kcal intake different from WT: *** *P* < .001. For panels (C) and (D), daily KO value different from WT value: *a*, *P* < .05; *b*, *P* < .01; *c*, *P* < .001. ^†^Statistical analysis by Mann–Whitney test. For (C), Day 1 KO value different from both Day -5 KO value and from Day 1 WT value: *d*, *P* < .001 by Kruskal-Wallis test followed by Dunn's multiple comparisons test for each comparison. A separate cohort of chow-fed male WT mice were individually housed and acclimatized on 10% LFD for 2 weeks and then were provided simultaneously with 60% HFD and 10% LFD admixed either with vehicle (10 mice) or LP-856866 at 75 mg/kg/day (10 mice): (E) daily intake of 10% LFD; (F) daily intake of 60% HFD; (G) daily total kcal intake; (H) change in BW from Day 0 value. For panels (E) through (H), LP-856866 daily kcal intake different from vehicle-treated group: *a*, *P* < .05; *b*, *P* < .01; *c*, *P* < .001. ^†^Statistical analysis by Mann–Whitney test. For panel (G), LP-856866 Day 1 kcal intake different from both Day 0 LP-856866 kcal intake and Day 1 Vehicle kcal intake: *e*, *P* < .001, and Vehicle Day 1 kcal intake different from Vehicle Day 0 kcal intake: *f*, *P* < .001, by Kruskal-Wallis test followed by Dunn's multiple comparisons test. Dotted line identifies 0 value at all time points.

When mice raised on chow and acclimated to LFD for 1 week were given a choice between LFD or 60% HFD, WT mice immediately preferred 60% HFD (Fig. 6A) but *Acsl5* KO mice lowered FC for 2 days and then preferred LFD, eventually approaching their prior level of LFD intake (Fig. 6B). Total kcal intake by KO mice was lower throughout the study (Fig. 6C), leading to a steadily increasing BW difference between KO and WT mice (Fig. 6D). The same response occurred when vehicle- or LP-856866-treated WT mice were given a choice between LFD or 60% HFD (Fig. 6E-6H). Of interest, total kcal intake significantly fell in KO but not WT mice on Day 1 of HFD exposure, and in LP-856866- but not vehicle-treated WT mice on Day 1 of treatment.

### Acsl5 KO Mice Have Increased Fecal FFAs

**Figure 7.**
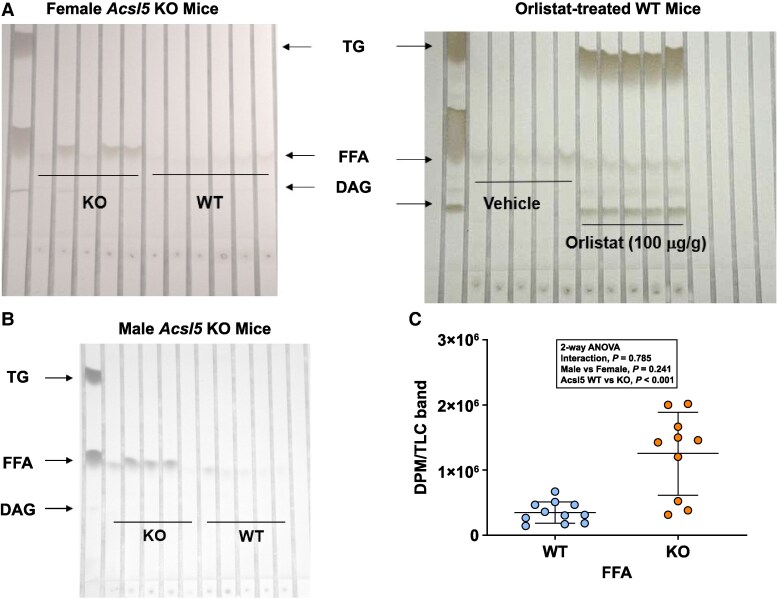
Effect of ACSL5 deficiency on stool FFA levels after an oral lipid load. (A) Left panel: chow-fed female *Acsl5* KO (n = 5) and WT (n = 6) mice underwent 24-hour stool collections that started immediately following an oral gavage of olive oil containing ^3^H triolein; lipids extracted from the stool collections were separated by TLC. Right panel: chow-fed adult male WT mice underwent 24-hour stool collections that started immediately following an oral gavage of olive oil containing either vehicle (n = 5) or 100 µg/g orlistat (n = 5); lipids extracted from the stool collections were separated by TLC. (B) Chow-fed male *Acsl5* KO (n = 5) and WT (n = 5) mice underwent 24-hour stool collections that were studied as described above for female mice. (C) TLC FFA bands from individual male and female *Acsl5* KO and WT mice were isolated and counted, followed by data analysis using 2-way ANOVA.

Decreased FC can explain the decreased body fat of ACSL5-deficient mice but cannot explain why, 6 hours after all mice received an equivalent oral ^3^H triolein TGLL challenge, ACSL5-deficient mice absorb 70% less TG than WT mice. To determine whether fat malabsorption might contribute, chow-fed *Acsl5* KO and WT mice, and WT mice treated with the lipase inhibitor orlistat, underwent 24-hour stool collections after receiving our standard TGLL challenge by oral gavage; ^3^H triolein was included in the olive oil given to *Acsl5* KO and WT mice. After separation of fecal lipids by TLC, orlistat-treated WT mice showed a marked increase in fecal fat, primarily in the TG fraction. In contrast, *Acsl5* KO mice showed a more modest increase in fecal fat, primarily in the FFA fraction (Fig. 7A and 7B). When the TLC fecal FFA bands were isolated and quantitated, *Acsl5* KO mice showed a 4-fold increase in ^3^H FFAs (Fig. 7C). In contrast, quantitation of TLC fecal TG bands from female mice showed much less ^3^H in TG bands than in FFA bands, with no difference in ^3^H TG between KO and WT mice (KO = 1.35 ± 0.44 × 10^5^ vs WT = 0.99 ± 0.25 × 10^5^ DPM/band, *P* = NS).

### ACSL5 Deficiency Delays Gastric Emptying by Activating the Ileal Brake

**Figure 8.**
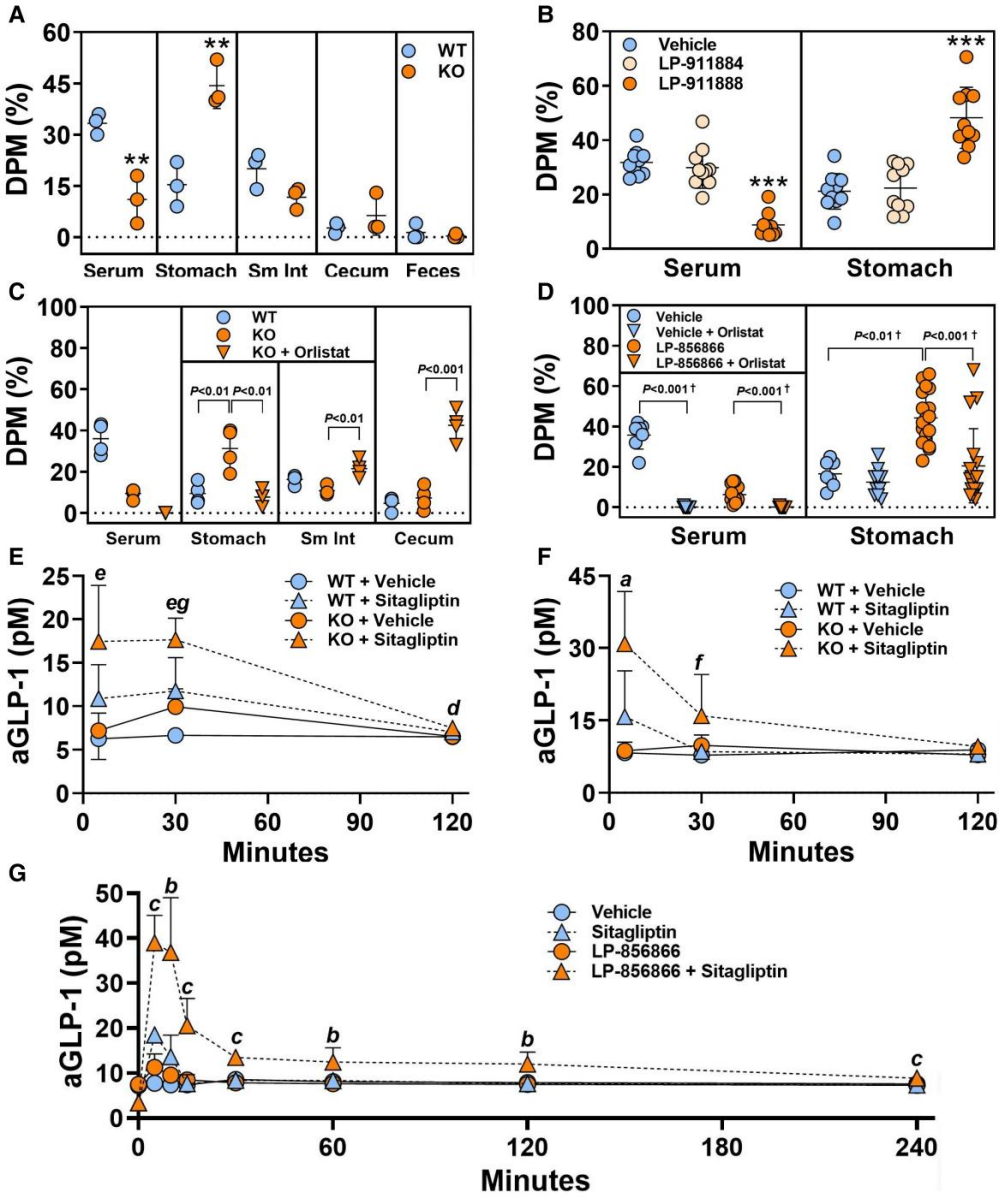
Effect of ACSL5 deficiency on gastric emptying and active GLP-1 levels. (A) Chow-fed male *Acsl5* KO (n = 3) and WT (n = 3) mice received IV tyloxapol followed by an oral gavage of olive oil containing ^3^H triolein. After 6 hours, blood, feces, and the indicated organs were collected and ^3^H activity was measured in each sample. KO different from WT, **P < .01. (B) Chow-fed male WT mice received IV tyloxapol followed by an oral gavage of olive oil containing ^3^H triolein and either vehicle (n = 10), 3 mg/kg LP-911884 (884; n = 10) or 3 mg/kg LP-911888 (888; n = 10). After 6 hours, blood and stomach were collected and ^3^H activity was measured in each sample. LP-911888 different from vehicle and from LP-911884 groups, ***P < .001. (C) Chow-fed male *Acsl5* KO (n = 8) and WT (n = 4) mice received IV tyloxapol followed by an oral gavage of olive oil containing ^3^H triolein and either vehicle (n = 4 KO mice, n = 4 WT mice) or 100 mg/kg orlistat (n = 4 KO mice). After 6 hours, blood, stomach, small intestine (Sm Int), and feces were collected and ^3^H activity was measured in each sample. (D) Chow-fed male WT mice received IV tyloxapol followed by an oral gavage of olive oil containing ^3^H triolein and either vehicle (n = 7), vehicle + 100 mg orlistat (n = 10), 30 mg/kg LP-856866 (n = 18), or both compounds (n = 10). After 6 hours, blood and stomach were collected and ^3^H activity was measured in each sample. ^†^Statistical analysis by Kruskal-Wallis test followed by Dunn's multiple comparisons test. For panels A-D, data reported as % of gavaged DPM. Chow-fed male (E) and female (F) *Acsl5* KO and WT mice (n = 5-10/group) were fasted overnight, pretreated with either vehicle (10% solutol) or sitagliptin (30 mg/kg) by oral gavage, and then provided a liquefied 45% HFD meal by oral gavage 30 minutes later. Plasma samples for aGLP-1 levels were obtained at 5, 30, and 120 minutes after administration of the 45% HFD meal. (G) Chow-fed male WT mice (n = 5/group) were fasted overnight, pretreated with either vehicle (10% solutol), 30 mg/kg sitagliptin, 60 mg/kg LP-856866, or both compounds by oral gavage, and then provided a liquefied 45% HFD meal by oral gavage 30 minutes later. Plasma samples for aGLP-1 levels were obtained at baseline and then at 5, 10, 15, 30, 60, 120, and 240 minutes after administration of the 45% HFD meal. Data in panels E-G were analyzed by 2-way ANOVA. Interaction between LP-856866 and sitagliptin: *a*, *P* < .05; *b*, *P* < .01; *c*, *P* < .001. Sitagliptin different from vehicle: *d*, *P* < .01; *e*, *P* < .001. KO different from vehicle: *f*, *P* < .05; *g*, *P* < .001.

The absence of marked fat malabsorption suggested that delayed GE contributed significantly to the slower rate of TG absorption during a TGLL challenge. This was confirmed by additional ^3^H triolein TGLL challenge studies showing that, 6 hours after oral delivery, ^3^H triolein was most abundant in the blood of WT mice and in the stomach of KO mice (Fig. 8A). GE was also delayed in mice pretreated with 3 mg/kg of the ACSL5i LP-911888 but not those pretreated with vehicle or 3 mg/kg LP-911884, which at this dose is the inactive enantiomer of LP-911888 (Figs. 3 and 8B). Because FFAs are generated by TG digestion before they are absorbed in the jejunum, the finding of excess FFAs in the feces of *Acsl5* KO mice implies that they also have excess ileal FFAs. Excess FFAs in the small intestine can activate the ileal brake, leading to decreased GE, gastrointestinal motility, and appetite. To confirm that excess intestinal FFAs triggered the delayed GE, we showed ^3^H triolein retention in the stomach of male *Acsl5* KO mice in the absence, but not presence, of orlistat, a lipase inhibitor that prevents intestinal FFA release by blocking TG digestion (Fig. 8C). This finding was confirmed by showing that the ACSL5i LP-856866 also led to gastric retention of ^3^H triolein in the absence, but not presence, of orlistat (Fig. 8D). Thus, the ^3^H triolein TGLL challenge study allowed us to observe the increased FFA and delayed GE that likely drive the thin phenotype of HFD-fed mice with ACSL5 deficiency.

### ACSL5 Deficiency Increases Intestinal GLP-1 Release

Because GLP-1 is one of the intestinal peptides linked to the ileal brake mechanism [32], we investigated aGLP-1 release in *Acsl5* KO mice. Chow-fed KO and WT mice were fasted overnight, pretreated with either vehicle or sitagliptin, which delays aGLP-1 inactivation, and then given a liquefied 45% HFD meal by oral gavage. For vehicle-treated males and females, plasma aGLP-1 levels were significantly higher in KO than WT mice 30 minutes after the HFD meal, with KO levels falling to WT levels by 120 minutes. At 5 and 30 minutes, aGLP-1 levels were even higher in sitagliptin-treated KO and WT mice relative to vehicle controls, but the highest levels were found in sitagliptin-treated KO mice (Fig. 8E-8F), where sitagliptin delayed aGLP-1 breakdown and allowed the marked local intestinal aGLP-1 release to be detected in systemic plasma samples. Based on 2-way ANOVA analysis, the combined effect of KO and sitagliptin on aGLP-1 levels was additive in males at 30 minutes and synergistic in females at 5 minutes. A similar pattern was seen when WT mice were pretreated with vehicle or with sitagliptin and/or LP-856866; the aGLP-1 increase was greater with sitagliptin than with LP-856866, but the 2 compounds together showed a synergistic increase in aGLP-1 that was still detected 4 hours after the HFD meal (Fig. 8G). Together, these studies showed a marked intestinal release of aGLP-1 by ACSL5-deficient mice after a HFD meal.

### ACSL5 Deficiency Is Associated With Improved Glucose Homeostasis

**Figure 9.**
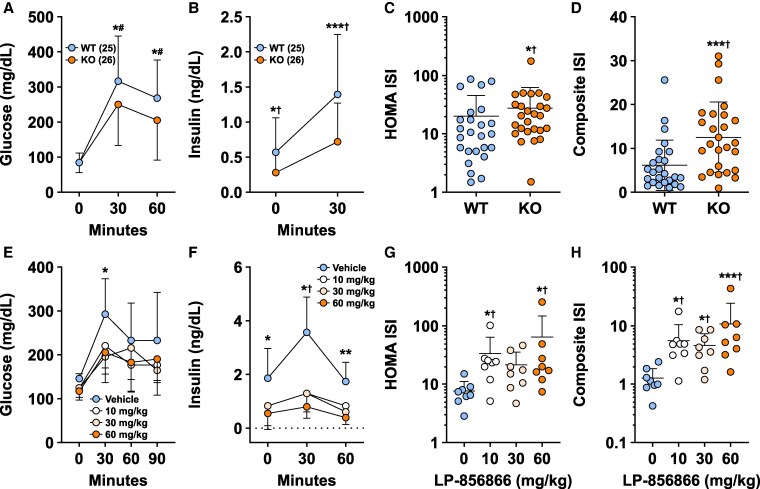
Effect of ACSL5 deficiency on glucose homeostasis and insulin sensitivity. Male and female *Acsl5* WT (n = 25) and KO (n = 26) mice maintained on 45% HFD for at least 13 weeks were fasted overnight and then underwent an OGTT: (A) Glucose excursion. (B) Insulin excursion. (C) HOMA ISI. (D) Composite ISI. KO mice different from WT mice: *P < .05; ***P < .001. ^†^Statistical analysis by Mann–Whitney test. #Compared normalized glucose values. The male DIO mice shown in Fig. 4 that received either vehicle or LP-856866 for 28 days (n = 8/group) were fasted overnight and then underwent an OGTT: (E) Glucose excursions; 30 mg/kg and 60 mg/kg groups different from vehicle-treated group, *P < .05. (F) Insulin excursions; all LP-856866-treated groups different from vehicle-treated group: *P < .05; **P < .01. ^†^Statistical analysis by Kruskal-Wallis test followed by Dunn's multiple comparisons test. (G) HOMA ISI and (H) Composite ISI; LP-856866-treated mice different from vehicle-treated mice: *P < .05; ***P < .001. ^†^ Statistical analysis by Kruskal-Wallis test followed by Dunn's multiple comparisons test.

In addition to the thin phenotype, multiple other favorable phenotypes were observed in mice with ACSL5 deficiency. OGTT glucose and insulin levels were lower in *Acsl5* KO mice (Fig. 9A and 9B), resulting in improved HOMA and composite ISIs (Fig. 9C and 9D). Similarly improved OGTT glucose and insulin levels (Fig. 9E and 9F) were seen in DIO mice after 28 days of LP-856866 treatment, leading to improved HOMA and composite ISIs (Fig. 9G and 9H).

### Less Liver Fat and Lower Serum Lipids in Acsl5-Deficient Mice

**Figure 10.**
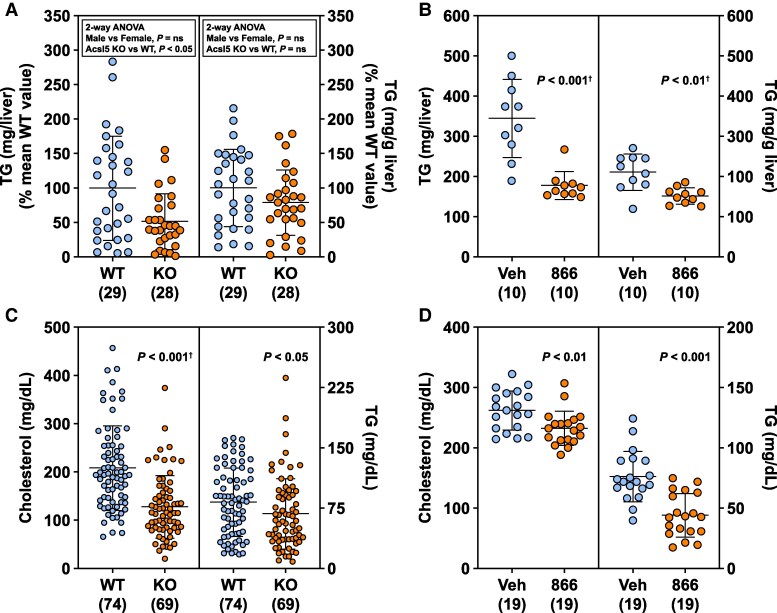
Effect of ACSL5 deficiency on liver fat and serum lipids. (A) Liver TGs, presented as either normalized mg/liver (left) or mg/g liver (right) were measured in *Acsl5* male (20 WT, 19 KO) and female (9 WT, 9 KO) mice. At time of harvest, the mice had been on 45% HFD for at least 5 months. ns, not significant. (B) Clinton 40% HFD-fed male mice were individually housed for 1 week and then divided into 2 groups of 10 mice each that received either vehicle (Veh) or 60 mg/kg LP-856866 (866) admixed in diet for 17 days. After an overnight fast, the mice had their livers harvested for hepatic TG levels, which are presented here as either mg/liver (left) or mg/g liver (right). (C) 45% HFD-fed male and female *Acsl5* KO and WT mice had serum levels of total cholesterol (left) and TG (right) measured after an overnight fast. (D) 45% HFD-fed male mice were individually housed for 1 week and then divided into 2 groups of 19 mice each that received either vehicle or 60 mg/kg LP-856866 admixed in diet for 3 weeks. Serum levels of total cholesterol (left) and TG (right) were measured after an overnight fast. ^†^Statistical analysis by Mann–Whitney test.

Liver fat, measured in nonfasted and fasted DIO *Acsl5* KO and WT mice maintained on 45% HFD for at least 5 months, was numerically lower in each subgroup of KO mice; when data from all KO and WT mice were normalized and pooled, liver fat was lower in KO mice (Fig. 10A). Liver fat was also lower in Clinton 40% HFD-fed WT mice treated with 60 mg/kg LP-856866 for 17 days (Fig. 10B). These findings are consistent with the lower liver histology fat scores observed in 2 additional cohorts of male DIO mice, 45% HFD-fed *Acsl5* KO mice (KO: 1.5 ± 1.3, n = 10 vs WT: 2.6 ± 0.7, n = 10; *P* < .05) and 45% HFD-fed WT mice treated with 60 mg/kg LP-856866 for 4 weeks (LP-856866: 2.1 ± 0.5, n = 15 vs vehicle: 2.9 ± 0.3, n = 17; *P* < .001). Serum total cholesterol and TG levels were lower in 45% HFD-fed *Acsl5* KO mice (Fig. 10C) and in 45% HFD-fed WT mice treated with 60 mg/kg LP-856866 for 3 weeks (Fig. 10D).

## Discussion

Mouse global gene KO phenotypes model with high fidelity both the phenotype of humans lacking function of the same gene and the effect of inhibitors targeting the protein product of that gene [13, 15, 33]. Our high-throughput screening of chow-fed global KO lines identified a favorable metabolic phenotype of decreased body fat, serum total cholesterol, serum TGs, and blood glucose, along with conserved LBM, in *Acsl5* KO mice, suggesting that systemic ACSL5 inhibition might produce the same favorable phenotype in men and women. Additional male and female mouse cohorts confirmed and extended these findings to show that the thin phenotype, although present in chow-fed KO mice, was exaggerated in HFD-fed KO mice due to decreased HFD intake and was accompanied by improved glucose tolerance and insulin sensitivity, and also by decreased liver fat and serum total cholesterol and TG levels.

Past work described global *Acsl5* KO mice as thin with increased energy expenditure and normal FC [10]. Recently, this same group reported that mice with an intestine-specific KO of *Acsl5* exhibit the same metabolic phenotype [12] as our global *Acsl5* KO mice. These findings are important from the drug discovery perspective; the favorable phenotype of global *Acsl5* KO mice suggests that the challenge of generating intestine-specific inhibitors [34] is not required, and that inhibiting ACSL5 beyond the intestine will not compromise the favorable phenotype. Also, the intestine-specific *Acsl5* KO model suggests that small molecule ACSL5i will not require central nervous system entry to inhibit FC, simplifying drug development.

TGLL studies using ^3^H-triolein showed that intestinal TG absorption was 70% lower in *Acsl5* KO mice, consistent with the 60% to 80% decrease in ACSL activity of KO small intestinal mucosa [10, 11]. We first identified LP-856866 and LP-911888 as potent small molecule ACSL5i, and we then used the ^3^H-triolein TGLL assay to show that the decrease in TG absorption was comparable between *Acsl5* KO mice and WT mice treated with these compounds. We then performed studies showing that, just as chow-fed KO mice decreased FC immediately after being switched to HFD, HFD-fed mice decreased FC immediately after receiving LP-856866 or LP-911888. Additional studies clarified how these compounds lowered FC. Although both compounds are potent inhibitors of ACSL5 and ACSL1, the ACSL most closely related to ACSL5 [35], our data suggest that the compounds work by inhibiting ACSL5 and not ACSL1 because LP-856866 decreased FC in WT but not in KO mice even though ACSL1 is expressed comparably by both WT and KO small intestinal mucosa. This is consistent with the finding that ACSL1 is a minor ACSL in mouse jejunal enterocytes [11]. Also, we used the enantiomers LP-911888 and LP-911884, which have large differences in ACSL5 inhibition but minimal structural differences, to show that the decrease in FC was greater with the more active enantiomer LP-911888. Our data suggest that changes in FC drive the body composition phenotype; changes in BW and body fat closely paralleled changes in HFD intake by *Acsl5* KO mice and by WT mice treated with LP-856866. The absence of differences in BW or body fat when intestine-specific *Acsl5* KO mice were pair-fed to WT mice further supports this conclusion [12].

Although systemic TG appearance was decreased over 6 hours in the TGLL bioassay, fecal TGs were not increased in *Acsl5* KO mice. Instead, KO mice had increased fecal FFAs after this oral TG challenge, implying an associated increase in ileal FFAs which is consistent with the presence of increased FFAs in the ileal mucosa of HFD-fed mice with an intestine-specific *Acsl5* KO [12]. This increase in ileal FFAs can activate the ileal brake, a term describing the distal to proximal feedback mechanism where exposing the ileum, the most distal part of the small intestine, to energy-containing nutrients generates signals that slow meal transit through the GI tract [32, 36]. Neural pathways have been linked to the ileal brake [32, 36, 37]. Also, gut peptides may participate; GLP-1 is released from the distal small intestine in response to a lipid load [38, 39] and high levels are associated with delayed GE [32, 36]. Recently, Zhang et al [37] showed that ileal GLP-1 activates local GLP-1R+ myenteric neurons which signal via abdominal sympathetic ganglia to gastric enteric neurons, resulting in gastric distention, and that this gastric distention then signals through spinal sensory neurons to neurons in the hypothalamus, resulting in food rejection but without any apparent effect on satiety. Importantly, they demonstrated that blocking these myenteric neurons completely disrupted the ileal brake mechanism, and their data support the hypothesis that the likely role of ileal GLP-1 release is to prevent nutrient malabsorption.

In animal and human studies, the most potent stimulus activating the ileal brake is delivery of TGs or FFAs to the ileum, which results in delayed GE and intestinal transit [32, 36, 40-42]; this is consistent with the delayed GE after an oral TG load observed here in *Acsl5* KO mice and in WT mice treated with an ACSL5i. TGs themselves did not stimulate the ileal brake mechanism in either dogs [43] or humans [38, 44] when co-administered with a lipase inhibitor that blocks intestinal lipase-mediated generation of FFAs from TGs, indicating that FFAs drive the ileal brake mechanism. This is consistent with absence of delayed GE in *Acsl5* KO mice, and WT mice treated with an ACSL5i, when the lipase inhibitor orlistat was added to the oral TG load. Ileal brake activation by delivering TGs or FFAs to the ileum results in less caloric intake in rodents and humans [32, 36, 38, 43-48], consistent with the lower caloric intake seen in HFD-fed *Acsl5* KO mice and WT mice treated with an ACSL5i. Even in our fat preference studies, the initial response of mice with genetic or pharmacologic Acsl5 deficiency was lower caloric intake, with LFD preference only appearing after 1 or 2 days. Importantly, the lower caloric intake associated with ileal brake activation in humans was not driven by nausea [38], consistent with evidence that ileal brake activation is meant to minimize nutrient malabsorption and does not involve central nervous system regions implicated in GLP-1 mimetic-induced nausea [37, 49, 50]. In contrast to the effect of ileal brake activation on caloric intake, the effect on satiety is less clear [32, 36, 38, 45, 48].

Human subjects responded to duodenal infusion of a TG emulsion with ileal brake activation and higher plasma GLP-1 levels but co-administering a lipase inhibitor blocked both GLP-1 release and ileal brake activation [38]. This is consistent with the higher aGLP-1 levels observed after an oral TG load in both *Acsl5* KO mice and WT mice treated with LP-856866. Although the aGLP-1 increase was not striking in ACSL5-deficient mice, co-treatment with the dipeptidyl peptidase 4 (DPP-4) inhibitor sitagliptin, which delays aGLP-1 breakdown, unmasked a marked intestinal release of GLP-1 that lasted for hours. These findings support data showing that intestinal GLP-1 acts locally to activate the ileal brake [37]. The ability of a GLP-1 receptor antagonist to partially reverse the decreased FC of HFD-fed mice with an intestine-specific *Acsl5* KO further supports a role for GLP-1 in ileal brake activation but also suggests that additional gut peptides and neural pathways may be involved in the ileal brake response to ACSL5 deficiency [12].

Past studies showed improved glucose tolerance and insulin sensitivity, and lower serum cholesterol and TG levels, in *Acsl5* KO models [10, 12]. Glucose tolerance and insulin sensitivity were also improved in our *Acsl5* KO mice and in LP-856866-treated WT mice. Fed TG levels were lower in our *Acsl5* KO mice and mice treated with LP-856866 as shown by the ∼70% fall in circulating ^3^H seen with the ^3^H triolein TGLL bioassay, and fasting TG levels were also lower in our ACSL5-deficient mice. Prior data in *Acsl5* KO models show that ACSL5 deficiency lowers liver fat burden [10, 12], consistent with our data in *Acsl5* KO mice and in LP-856866-treated DIO mice; hepatic ACSL1 inhibition by LP-856866 is unlikely to contribute to the lower liver fat burden because mice with a liver-specific *Acsl1* KO were not protected from HFD-induced hepatic steatosis [51]. Finally, consistent with findings in mice with an intestine-specific *Acsl5* KO [12], total cholesterol levels were lower in our ACSL5-deficient mice.

In enterocytes and hepatocytes, TGs are assembled by the sequential activity of monoacylglycerol and diacylglycerol acyltransferases (MGAT and DGAT) and then packaged into chylomicrons and very low-density lipoprotein, respectively, by microsomal TG transfer protein (MTP) [52]. MTP is not redundant. The *Mtp* KO is embryonic lethal, while *Mtp* HET mice and humans with inactivating MTP mutations have low circulating lipid levels, hepatic steatosis, and fat malabsorption with diarrhea when on a HFD [53, 54]. Lomitapide, which inhibits MTP systemically, lowers cholesterol and TGs in people with homozygous familial hypercholesterolemia, but dosing must be titrated to avoid hepatic steatosis and steatorrhea [55, 56]. In rodent enterocytes, the intestine-specific MTP inhibitor JTT-130 blocks chylomicron generation which increases fecal TGs [57], consistent with findings in people with MTP mutations or taking lomitapide, but JTT-130 also exactly reproduced the *Acsl5* KO metabolic phenotype, including decreased liver fat in DIO rats [57-63]. This *Acsl5* KO metabolic phenocopy is also present in mice with genetic or pharmacologic inhibition of DGAT1 and MGAT2 which, unlike MTP, have compensatory pathways driving TG assembly in their absence [52, 64-66]. These data suggest that this common metabolic phenotype is driven by dietary FFAs that activate the ileal brake after accumulating in the small intestine due to impaired upstream TG re-esterification.

In humans, DGAT1 is not redundant; DGAT1 deficiency or inhibition results in severe diarrhea [52]. In contrast, human MGAT2 is redundant, and the MGAT2 inhibitor BMS-963272 was generally well-tolerated while lowering BW and increasing GLP-1 levels during a 14-day study of people with obesity [66]. ACSL5 is also redundant, as exemplified by TGLL data showing that absorption of an oral TG bolus by *Acsl5* KO mice was 30% of WT values. This redundancy resulted in no increase in fecal TG and a 4-fold increase in fecal FFA over 24 hours after a standard oral TG load; the fecal FFA increase was qualitatively modest compared to the increased fecal TG of orlistat-treated mice after the same TGLL challenge. Because the lipids were delivered by oral gavage, *Acsl5* KO mice could not regulate their fat intake; the TGLL challenge allowed us to understand the mechanism behind their avoidance of HFD but likely resulted in more intestinal FFA than occurs when KO mice can regulate intake. Regardless, our *Acsl5* KO cohorts exhibited steady BW gain without diarrhea when switched to HFD at weaning, suggesting that the redundant pathway allows most dietary TG to be absorbed by HFD-fed *Acsl5* KO mice, consistent with findings in HFD-fed mice with an intestine-specific *Acsl5* KO [12]. Thus, inhibiting the TG re-esterification pathway at the level of ACSL5 has the potential to improve hepatic steatosis while minimizing diarrhea risk.

Just as in mice, ACSL5 is the predominant ACSL expressed in human small intestine but is a minor ACSL in liver, where ACSL1 predominates (https://gtexportal.org/home/). Genome-wide association study (GWAS) associations with the human ACSL5 gene are strong for BW and serum levels of aspartate aminotransferase, TG and total cholesterol, and are moderate for liver fat content, A1c, and serum levels of alanine transaminase (https://hugeamp.org, September 18, 2025). Also, a strong type 2 diabetes GWAS locus is located within the *TCF7L2* gene, with intronic single nucleotide polymorphism rs7903146 the likely causal variant [67]. Deleting this region markedly lowered *ACSL5* expression and protein levels [68], leading to the current hypothesis that ACSL5 participates in the mechanism that influences diabetes risk [67]. Although we did not observe diarrhea in *Acsl5* KO mice, 6 neonates in one consanguineous family homozygous for an inactivating *ACSL5* mutation had diarrhea and vomiting requiring parenteral nutrition [69]. Importantly, these children developed normally after the neonatal period without dietary or other restrictions suggesting that severe GI effects will be unlikely in adults receiving an ACSL5i, a conclusion supported by the fact that the MGAT2 inhibitor BMS-963272, which targets the same pathway, was generally well-tolerated in humans [66].

Our study has limitations. ACSL5 inhibition has yet to be shown to promote BW loss in humans; however, the thin phenotypes of *Cnr1* and *Mgat2* KO mice model the BW loss of humans receiving the CNR1 inhibitor rimonabant and the MGAT2 inhibitor BMS-963272 [64-66, 70, 71], and MGAT2 is in the same pathway for TG reassembly as is ACSL5, suggesting that ACSL5 inhibition will likely result in human BW loss. Also, the compounds presented here are dual ACSL5 and ACSL1 inhibitors that were used solely to test if ACSL5 inhibition reproduced the *Acsl5* KO metabolic phenotype because mouse *Acsl1* KO data indicate that long-term ACSL1 inhibition adversely affects muscle function [72, 73], thus signaling the need to develop highly selective ACSL5i for human use. Finally, although intestine-specific *Acsl5* KO mice showed postprandial increases in both GLP-1 and peptide YY (PYY) levels [12], we did not explore the role of PYY in FFA-mediated ileal brake activation in our genetic and pharmacologic models of ACSL5 deficiency.

In summary, we validated our novel ACSL5i by showing that pharmacologic inhibition replicates the favorable metabolic phenotype of decreased body fat, liver fat, insulin sensitivity, and serum lipid levels, along with improved glucose tolerance, that is found in HFD-fed *Acsl5* KO mice. We also showed that combining ACSL5 deficiency with HFD feeding activated the ileal brake, resulting in the delayed GE and decreased FC that drove this phenotype. We expect, based on GWAS data and recent studies with an MGAT2 inhibitor, that selective ACSL5 inhibition is likely to reproduce in humans some or all of the favorable metabolic phenotype described above for ACSL5-deficient mice.

## Data Availability

Data will be made available upon reasonable request.

## Abbreviations

ACS: acyl-coenzyme A synthetase
ACSL: long-chain acyl-coenzyme A synthetase
ACSL5i: acyl-coenzyme A synthetase long-chain family member 5 inhibitor
aGLP-1: active glucagon-like peptide 1
BW: body weight
CoA: coenzyme A
DAG: diacylglycerol
DGAT: diacylglycerol acyltransferase
DIO: diet-induced obesity
DPM: disintegrations per minute
FC: food consumption
FFA: free fatty acid
GE: gastric emptying
GI: gastrointestinal
GIP: gastric inhibitory polypeptide
GLP-1: glucagon-like peptide 1
GLP-1R: glucagon-like peptide 1 receptor
GWAS: genome-wide association study
HET: heterozygous
HFD: high-fat diet
HOMA: homeostatic model assessment
HTPS: high-throughput phenotypic screen
ISI: insulin sensitivity index
KO: knockout
LBM: lean body mass
LCFA: long-chain fatty acid
LFD: low-fat diet
MGAT: monoacylglycerol acyltransferase
MTP: microsomal triglyceride transfer protein
OGTT: oral glucose tolerance test
PCR: polymerase chain reaction
TG: triglycerides
TGLL: tyloxapol gastric lipid loading
WT: wild-type
